# Mental Health of Prison Inmates During the COVID-19 Pandemic: A Systematic Review

**DOI:** 10.3389/ijph.2024.1607166

**Published:** 2024-11-21

**Authors:** Francisco Javier González-Riera, Juan Jesús García-Iglesias, Regina Allande-Cussó, Carlos Ruiz-Frutos, Luciano Rodríguez-Diaz, Juana María Vázquez-Lara, Francisco Javier Fernández-Carrasco, Javier Fagundo-Rivera, Juan Gómez-Salgado

**Affiliations:** ^1^Department of Health Education and Community Participation, Ministry of Health, Jaén and Jaén Sur Health District, Andalusian Health Service, Seville, Spain; ^2^Department of Sociology, Social Work and Public Health, Faculty of Labour Sciences, University of Huelva, Huelva, Spain; ^3^Department of Nursing, Faculty of Nursing, Physiotherapy and Podiatry, Sevilla University, Seville, Spain; ^4^Safety and Health Postgraduate Programme, Escuela de Posgrado, Universidad de Especialidades Espíritu Santo, Guayaquil, Ecuador; ^5^Department of Nursing, Faculty of Health Sciences of Ceuta, University of Granada, Granada, Spain; ^6^ Centro Universitario de Enfermería Cruz Roja, Adscrito a la Universidad de Sevilla, Sevilla, Spain

**Keywords:** psychological distress, fear, prisons, mental health, anxiety

## Abstract

**Objectives:**

The aim of this study was to comprehensively assess how COVID-19 affected the levels of different mental health variables in prison inmates.

**Methods:**

A systematic review was conducted following the PRISMA format in the Pubmed, Scopus, Web of Science, PsycINFO, and ScienceDirect electronic databases between August and September 2023. Methodological quality was assessed using the critical appraisal tools for studies of the Joanna Briggs Institute.

**Results:**

Thirteen studies were included. The studies found increased levels of stress, anxiety, fear, depression, and negative emotions associated with lack of information about the pandemic and isolation leading to reduced social interaction. In addition, lack of access to common recreational spaces, limited access to support resources, especially mental health resources, fear of contracting the virus, and lack of trust in prison staff and in themselves to be protected were identified.

**Conclusion:**

Further research may be necessary in prison populations with added vulnerability, such as the elderly, women, transgender and non-binary persons, to determine specific interventions, after assessing the prevalent psychological sequelae. Prevention strategies and mental health promotion are also encouraged.

## Introduction

The COVID-19 pandemic, hereafter referred to as the pandemic, has had a significant impact on the physical and mental health of the prison population, as a particularly vulnerable group [1]. This was due to pre-existing prison conditions (e.g., overcrowding) [2], highlighting poor ventilation [3], inadequate resources and healthcare [4], and to many inmates having pre-existing physical and mental health needs [5].

The main difficulties encountered during imprisonment, besides the pandemic, include: rejection by society, placing the prisoner in an inferior social category, being under the influence of other people, and being deprived of autonomy, facilities, services, sexual contact, or a sense of security. These difficulties were exacerbated by the disruption of their way of life, particularly in terms of family contact, occupational, leisure and social activities, decision-making opportunities, and by the lack of privacy in their shared living space. This constant deprivation of basic needs can lead to frustration, conflicts, negative experiences, and poor mental health [6]. The prison system differs across countries. The majority of prisoners globally are men (93%), but over the last 20 years the number of women in prison has shown a greater increase (33% increase) than that of men (25%). There are large differences in incarceration rates across regions, ranging from almost 600 inmates per 100,000 inhabitants in North America, to around 50 in South Asia. In 2019, the global mean was estimated at 152 incarcerated persons per 100,000 population. Over the last 20 years, North America, Sub-Saharan Africa, and Eastern Europe have reduced long-term imprisonment rates -up to a 27% decrease since 2000- but Latin America and Australia/New Zealand have increased them -up to 68%. Prisoners in half of the countries are in overcrowded facilities, at more than 100% of capacity [1].

As social beings, people need interaction, which has proven positive effects on the mental health [7, 8]. Conversely, social isolation can have long-lasting negative consequences [8–10], especially in vulnerable populations [11]. In the long term, solitary confinement, referring to the physical and social isolation of an individual, is considered torture [12] and is associated with psychological consequences such as increased levels of anxiety and stress, sleep disruption, loss of emotional control, paranoia, cognitive disturbances, and increased self-harm, suicide [8, 9], depression, and aggression [8, 13].

Following recent infectious outbreaks such as SARS, Influenza A/N1H1, and Ebola, evidence has emerged on the impact of such threats on psychological wellbeing [14]. In this line, greater psychopathological morbidity, primarily anxiety and depression, has been identified during early stages of these outbreaks [14–17]. In this sense, there is evidence of the influence that information has had on reactions of fear, anxiety, and depression, as well as on the application of adaptive protective behaviours [16, 18, 19].

The development of protective behaviours, such as avoiding social contact or adopting hygiene patterns, has been described as related to the amount of information received, to individual attitudinal factors, and to levels of anxiety. Among the attitudinal factors, perceived susceptibility, perceived severity of the disease, and assumptions about the efficacy of the recommended behaviours were highlighted [15, 16, 18, 19]. When describing the relationship between protective behaviours and anxiety and depression, it is generally described that higher levels of anxiety are associated with more appropriate and consistent protective behaviours [15, 20], although other studies have found opposing data [18, 21]. And regarding the information received, it must be clear and consistent in order to encourage the implementation of protective behaviours [19–22].

From here, in the early phases and during the quarantine period of the COVID-19 pandemic in 2020, research was developed on the psychological effects on emotional wellbeing and the sense of coherence [20, 23, 24]. By integrating data on the impact of other outbreaks, evidence suggested that a large-scale lockdown would lead to a range of psychological consequences. Early data from the epidemic originating in China indicated the importance of protecting mental health, noting high levels of anxiety. In addition, protective factors such as adequate health information and protective measures were identified [21]. On the other hand, sadness, grief, and fear have been reported as emotional reactions. The relationship between fear and the high rates of infection, morbidity, and mortality associated with the disease has been described. Thus, high levels of fear can lead to maladaptive behaviours and emotional responses [25]. The Fear of COVID-19 Scale (FCV-19S) was validated in English to measure the level of fear [26], as well as in Italian [27] and Spanish [25].

Limited published research has identified that isolation and anxiety related to the risk of infection were common issues in all studies examining the impact of COVID-19 on the mental health of imprisoned populations [28]. The adverse effect of fear of COVID-19, the effects of social distancing and isolation, and the disruption of visits, as well as the reduction or discontinuation of mental health services, are described as having a detrimental effect on the mental wellbeing of inmates [29]. Finally, social isolation in prisons has been identified as aggravating prisoners’ mental health problems and even suicide rates, and poor communication about the pandemic by prison staff is reported to contribute to increased stress and anxiety among inmates [30]. It should be noted that, in the pandemic, isolated prisoners often spent their time in a single cell for 22.5–24 h a day and that solitary confinement could last for months or years, for an indeterminate length of time [31].

In conclusion, and in accordance with the above, this study is considered useful in order to describe how COVID-19 has affected the levels of different mental health variables in prison inmates. In addition, as a secondary and/or derived objective, the aim is to identify predisposing and precipitating factors of the psychological consequences of the pandemic, always with the goal of improving the public health of the inmate population, especially their mental health.

## Methods

### Study Design

A systematic review was conducted following the guidelines of the PRISMA (Preferred Reporting Items for Systematic Reviews and Meta-Analyses) statement [32]. The protocol followed is listed in the International Prospective Register of Systematic Reviews (PROSPERO) [33] with code CRD42023492481.

### Databases and Search Strategy

The search was carried out in the Pubmed, Scopus, Web of Science, and PsycINFO electronic databases on the basis of the key words that the research question yielded following the Condition, Context, Population strategy (CoCoPop) [34] (Table 1).

**TABLE 1:** CoCoPop format: key words (COVID-19 in Prisons, Spain, 2020–2024).

Condition	Mental health levels
Context	In prisons during COVID-19
Population	Prison inmates
Research question	How does COVID-19 affect mental health levels in prison inmates?

Based on these key words, the *Medical Subject Headings* (MeSH) thesaurus was consulted, yielding the following descriptors: *prison*, *mental health*, and *COVID-19.* In order to increase the number of published studies in line with the subject of the study, related terms capable of completing the search strategy were used to supplement the search strategy based on the MeSH descriptors (Supplementary File 1), which were joined using the Boolean operators *AND* and *OR.*

In Table 2, the search strategy used for each of the mentioned databases during the search process on 29 September 2023 is listed.

**TABLE 2:** Search strategy (COVID-19 in Prisons, Spain, 2020–2024).

Database	Search strategy	Results
Pubmed	(((((((mental health[Title/Abstract] OR depression[Title/Abstract] OR anxiety[Title/Abstract] OR stress[Title/Abstract] OR fear[Title/Abstract] OR burnout[Title/Abstract]) OR (mental health[MeSH Terms])) OR (anxiety[MeSH Terms])) OR (Psychological Distress[MeSH Terms])) OR (depression[MeSH Terms])) OR (Psychological Burnout[MeSH Terms])) AND ((COVID-19[Title/Abstract]) OR (COVID-19[MeSH Terms]))) AND ((Prison*[Title/Abstract] OR Penitentiar*[Title/Abstract] OR inmates[Title/Abstract] OR criminals[Title/Abstract] OR offenders[Title/Abstract] OR “incarcerated people”[Title/Abstract]) OR (prisons[MeSH Terms]))	100
Scopus	(TITLE-ABS-KEY (prison* OR penitentiar* OR inmates OR criminals OR offenders OR “incarcerated people”) AND TITLE-ABS-KEY (“mental health” OR depression OR anxiety OR stress OR fear OR burnout OR “Psychological Distress” OR “Psychological Burnout”) AND TITLE-ABS-KEY (COVID-19) )	304
Web Of Science	Prison* OR Penitentiar* OR inmates OR criminals OR offenders OR “incarcerated people” (Topic) AND “mental health” OR depression OR anxiety OR stress OR fear OR burnout OR “Psychological Distress” OR “Psychological Burnout” (Topic) AND COVID-19 (Topic)	297
PsycInfo	tiab(Prison* OR Penitentiar* OR inmates OR criminals OR offenders OR “incarcerated people”) AND tiab(“mental health” OR depression OR anxiety OR stress OR fear OR burnout OR “Psychological Distress” OR “Psychological Burnout”) AND tiab(COVID-19)	69
Other sources	Items identified through other resources	5
Date of search: 29 September 2023	Total	775

### Selection Criteria

For the selection of the articles, the following criteria were used:

#### Inclusion Criteria

• Original articles published in English, Spanish, French, and Portuguese.
• Type: original articles, short communication, and case reports.
• Articles dealing with imprisoned or recently released individuals, if they discussed their period of imprisonment during the COVID-19 pandemic.
• Population: being an adult/≥18 years in prison.
• Articles measuring any of the following values and/or effects: mental health; stress; distress; anxiety; fear; depression; mood; self-esteem; confidence; psychological pain; suicide during the COVID-19 pandemic; mental health differences between sexes or sexual orientation; predisposing and precipitating factors of the psychological consequences of the COVID-19 pandemic.

#### Exclusion Criteria

• Studies in languages other than English, Spanish, French, and Portuguese.
• Type: opinion articles, editorials, and letters to the editor/publisher.
• Population: <18 years in juvenile detention centres.
• Studies of low scientific-technical quality after applying the quality assessment tool.
• Articles that did not answer the research question and were not related to the objective of the review.

### Data Collection and Extraction

Two researchers independently performed searches, eliminated duplicate studies, and selected articles for inclusion after reading the abstract and title, according to the predetermined criteria. The same two authors then reviewed the full text of the potentially eligible articles, and the decision to include or exclude studies from the review was made by consensus. Any discrepancies were resolved by a third author. A snowball search of the study references yielded 5 potentially eligible studies.

A narrative synthesis of the findings will be conducted. Popay et al.’s guidelines for conducting a narrative synthesis will be followed in this review [35]. This framework consists of four main elements: 1) developing a theory of how prisoners’ mental health has been affected during the COVID-19 pandemic; 2) developing a preliminary synthesis of the findings obtained from the included studies; 3) exploring relationships in the data; and 4) assessing the robustness of the synthesis. Firstly, descriptive summaries were drafted for each of the studies including all of the relevant information from the data extraction sheet. The step included tabulation of extracted data including details of author and year of publication, country, design and objective, participants, instrument, and main results; in addition, the results of the JBI critical appraisal tool were added, which also helped to provide a preliminary exploration of the relationships within and across studies. By using a thematic analysis, the main, recurring, and most prominent issues across multiple studies were systematically identified. Subgroups were then created based on levels of anxiety, depression, stress, fear, and other data such as the instruments used, socio-demographic data, emotional disorders, coping strategies, predisposing and precipitating factors of the psychological consequences of the COVID-19 pandemic, sex differences or sexual orientation, etc.

### Methodological Quality Assessment

Two reviewers independently determined the methodological quality of the selected studies using the critical appraisal tools for studies of the Joanna Briggs Institute (JBI) at the University of Adelaide [36]. These tools allow to assess the methodological quality of a study and to determine the extent to which a study has excluded or minimised the possibility of bias in its design, conduct, and/or analysis. The versions for cross-sectional quantitative studies (8 items) and for qualitative studies (10 items) were used, setting the cut-off point at 6 to be accepted for inclusion in this review (Tables 3, 4).

**TABLE 3:** Scores of cross-sectional observational studies (COVID-19 in Prisons, Spain, 2020–2024).

Study	JBI	The participants and the environment are described in detail	Inclusion criteria are clearly defined	Exposure was validly and reliably measured	The criterion used to measure the condition was objective	Confounding factors were identified	Strategies for dealing with confounding factors	Valid and reliable measured results	Appropriate statistical analysis was used
Birkie et al. [37]	7/8	YES	YES	YES	YES	NO	YES	YES	YES
Chimicz et al. [38]	6/8	YES	YES	YES	YES	NO	N/A	YES	YES
DePalma et al. [39]	7/8	YES	YES	YES	YES	YES	NO	YES	YES
Di Giuseppe et al. [40]	7/8	YES	YES	YES	YES	YES	NO	YES	YES
Esteban-Febres et al. [41]	6/8	YES	YES	YES	YES	NO	NO	YES	YES
Kothari et al. [42]	6/8	YES	YES	YES	YES	NO	NO	YES	YES
Mendes et al. [43]	7/8	YES	YES	YES	YES	YES	NO	YES	YES
Zhang et al. [44]	6/8	YES	YES	YES	YES	N/A	N/A	YES	YES

Unclear or Not Applicable, N/A.

**TABLE 4:** Scores of qualitative studies (COVID-19 in Prisons, Spain, 2020–2024).

Study	JBI	Congruence between stated philosophical perspective and research methodology	Congruence between methodology and question/objectives	Congruence between the methodology and the method used to collect the data	Congruence between methodology and data representation and analysis	Congruence between methodology and interpretation of results	Cultural and theoretical localisation	Influence of the researcher on the sample and *vice versa*	Representativeness of participants	Ethical approval by an appropriate body	Relationship between findings and analysis or interpretation of data
James et al. [45]	9/10	YES	YES	YES	YES	YES	YES	YES	N/A	YES	YES
Montagnet et al. [46]	7/10	YES	YES	YES	NO	YES	YES	YES	NO	N/A	YES
Sorge et al. [47]	7/10	YES	YES	YES	NO	YES	YES	YES	NO	N/A	YES
Suhomlinova et al. [48]	9/10	YES	YES	YES	NO	YES	YES	YES	YES	YES	YES
Wainwright et al. [49]	8/10	YES	YES	YES	YES	YES	NO	YES	NO	YES	YES

Unclear or Not Applicable, N/A.

## Results

**FIGURE 1:**
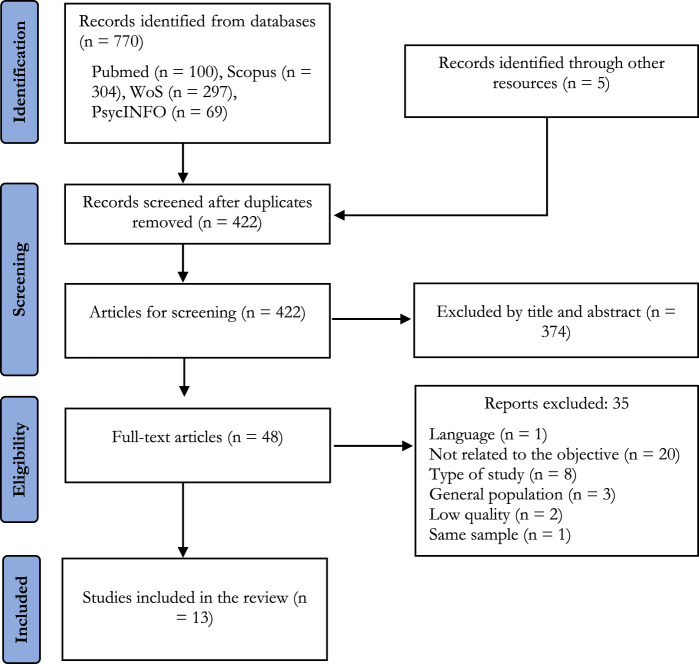
Study flowchart (COVID-19 in Prisons, Spain, 2020–2024).

The initial search strategies identified a total of 430 references, which were then screened according to the topic of this review. A total of 13 studies were finally selected (Figure 1), 8 of which were quantitative and 5 qualitative.

Three articles were found in the United States [39, 45, 46], two in Italy [40, 47], one in Ethiopia [37], one in Peru [41], one in Portugal [43], one in China [44], one in Poland [38], and three in the United Kingdom [42, 48, 49].

Anxiety was studied in 10 of the 13 articles included [37–39, 41, 43–46, 49]; depression and mood in 9 [37–41, 43–45, 49] predisposing and precipitating factors in 7 [37, 42, 43, 45, 46, 48, 49]; cognitions and complex emotions in 8 [37, 38, 40, 42, 43, 46, 47, 49]; basic emotions in 6 [38, 39, 41, 43, 47, 49]; stress in 5 [38, 39, 41, 43, 48]; differences in mental health between sexes or by sexual orientation in 4 [41, 43, 45, 48]; and suicide in 2 [41, 48].

In 10 of the 13 articles selected, the sample consisted only of prison inmates, of which 2 were women and 1 article included transgender women and non-binary persons. Two articles included recently released persons, both men and women, who were surveyed about their experience in prison.

Finally, one of the included articles also surveyed health professionals in addition to the inmates, although only the inmates’ responses were included in the differentiated results.

After applying the JBI critical appraisal tool, medium-high scores were obtained in both cross-sectional observational studies and qualitative studies.

By country, it was found that the factors studied differed across studies. Among the countries from which only one study was included, in Ethiopia [37], the factors assessed were depression, anxiety, and associated factors. In Poland [38], changes and stressors related to COVID-19 were studied, which moods and emotions were most common after the pandemic, and which of the selected factors determined the positive and negative mood of inmates. In Peru [41], levels of psychological distress among female inmates were studied. In Portugal [43], perceptions of fear of COVID-19 and the psychological impact of the pandemic were assessed in a sample of young adult male inmates. In China [44], anxiety levels were compared across inmates pre- and post-COVID-19, and the causes of changes in anxiety levels were analysed.

Among the countries where several studies were included, in Italy [40, 47], knowledge, attitudes, and preventive practices towards COVID-19 were assessed from the perspective of incarcerated persons. Also, the study explored the psychological effects of confinement during the early stages of the pandemic.

In the United States [39, 45, 46], anxiety symptoms, depressive symptoms, and self-rated health were compared before and during the pandemic. Another study described the COVID-19 risk mitigation strategies implemented and their impact on the mental health of incarcerated women. In addition, another study explored the changing conditions of confinement in the early months of the pandemic, as well as how incarcerated persons experienced the pandemic in terms of mental and physical health, safety, and trust in correctional institutions. Studies in the UK [42, 48, 49] assessed whether there had been changes in mental health as a result of the pandemic and what this had contributed to, the understanding the impact of reduced routines and other changes. Also, whether those who required mental health support felt supported by the minimum mental health services in the prison. Another study explored how a socially excluded gender, the transgender and non-binary (TGNB) minority group, experienced and coped with the stressors of the pandemic. Another study assessed how healthcare in prisons had changed in response to COVID-19 and how these changes were experienced.

Finally, only in Peru, Portugal, the US, and the UK were sex/gender differences specifically analysed.

Table 5 shows the characteristics of each of the 13 studies included in this review.

**TABLE 5:** Characteristics of the studies included in the systematic review (COVID-19 in Prisons, Spain, 2020–2024).

Studies	Context	Objective of the study	Type of study	Participants	Methods and instruments	Main findings	Quality of studies
Birkie et al. [37]	Ethiopia	To assess depression, anxiety, and associated factors among Dessie City prisoners during the 2020 COVID-19 outbreak	Quantitative cross-sectional study	Dessie’s correctional prisoners (n = 420) Mean age: 29.79 Males: 79.3% Females: 20.7%	- PHQ-9 - GAD-7 - Brief COPE - Oslo 3-item social support scale - Insomnia Severity Index	The overall prevalence of depression and anxiety was significantly high, and was related to a number of factors including COVID-19 Of the 420 study participants, 279 (66.4%) had major depressive disorder with 95% CI of (61.4%, 70.6%) 281 (66.9%) had generalized anxiety disorder with a 95% CI of (61.9%, 71.9%) Factors associated with Anxiety were age, where those with ages from 18 to 25 to 26–33 were 81% and 82% less likely to have generalised anxiety disorder, respectively, than those 42 years old or older. Those who stayed in prison for 1–2 years were almost twice as prone to generalised anxiety disorder [AOR = 95% CI: 1.87 (1.05, 3.35) and 1.89 (1.01, 3.55)] than those with a stay of less than 1 year. Those having high worry, while showing symptoms associated with COVID-19 and a long stay in the prison were almost twice as prone to generalised anxiety disorder [AOR = 95% CI: 1.87 (1.05, 3.35) and 1.89 (1.01, 3.55)] than those having moderate worry and a prison stay of 1 year or less	7/8
Chimicz et al. [38]	Poland	To find out (a) how challenging for inmates were the selected COVID-19 pandemic-related changes and stressors, (b) what moods and emotions are most commonly experienced by prisoners just after the difficult period of the COVID-19 pandemic, and (c) which of the selected factors determine the positive and negative mood of inmates	Quantitative cross-sectional study	Prisoners in six Polish prisons (n = 250) Mean age: 64% 25–45 Males: 100% Females: 0%	- General Mood Scale - Mood Scale (positive and negative) - Emotions Questionnaire	A depressed mood predominated among the prisoners, making them feel unhappy, discouraged, tense, and uptight. Dominant feelings of alienation, distress, anxiety, and worry were reported For positive moods in inmates who were not infected with COVID-19, joy, angst, and contentment appeared to be significant predictors. The more strongly inmates experienced joy and contentment and the less angst, the more positive their mood. It was also found that the weaker the sadness they experienced, the stronger the tendency for them to experience positive mood (*p* = 0.058) A statistically significant model was obtained for inmates who got sick with COVID-19 (F (2,109) = 8.091; *p* < 0.001). The variables included in the model together explain 62% of the variance. Happiness appeared to be the only significant predictor. Inmates experienced a more positive mood along with experiencing happiness. In addition, a tendency for improved mood was found to occur with a stronger experience of a slump (*p* = 0.057) The feeling of joy appeared to be a significant predictor of negative mood for those inmates who had no personal experience with COVID-19 It is necessary to provide convicts with continuous psychological care and to monitor their mood. Such measures should be the foundation for restorative interventions	6/8
DePalma et al. [39]	United States	To compare anxiety symptoms, depressive symptoms, and self-assessed health, before and during the pandemic	Quantitative longitudinal study	Incarcerated persons ≥50 years (n = 157) Mean age: 56 Males: 96% % Females: 4%	- PHQ-8 - GAD-7 - SRH	From before to during the pandemic - Anxiety symptoms increased (from 6.4 ± 5.7 to 7.8 ± 6.6; *p* < 0.001) - Depressive symptoms increased (from 5.5 ± 6.0 to 8.1 ± 6.5; *p* < 0.001) and self-assessed health decreased The mediation model indicated that worsening (higher) anxiety led to worsening (higher) depressive symptoms	7/8
Di Giuseppe et al. [40]	Italy	To assess knowledge, attitudes, and preventive practices toward COVID-19 disease of incarcerated people	Quantitative cross-sectional study	Inmates in three prisons in the geographic area of Campania region (n = 685) Mean age: 42.4 Males: 100% Females: 0%	- PHQ-2 - GAD-2	High level of anxiety and depression in a proportion of the study population: more than half (51.7%) and almost half (45.3%) were screened positive for symptoms of generalized anxiety and depression, respectively 85.4% believed that COVID-19 could cause serious consequences in their institution Only 22.6% were self-confident about their ability to protect themselves from SARS-CoV-2 infection. This attitude was significantly higher in those who were involved in working activities in the institution	7/8
Esteban-Febres et al. [41]	Peru	To establish the levels of psychological distress amongst female inmates at a high-security prison in Lima in November 2020	Cross-sectional descriptive study	Inmates at the Women’s Prison of Chorrillos, Lima (n = 314) Mean age: 40 Males: 0% Females: 100%	- BSI-18	Results showed that 34.6% of the inmates could be considered as cases of psychological distress The depression subscale had the highest score, followed by the anxiety and the somatisation subscales The most prevalent symptom was “feeling blue” The most common symptoms included “feeling lonely”	6/8
James et al. [45]	United States	To describe the COVID-19 risk mitigation strategies implemented in California prisons and the impact of these policies on the mental health of incarcerated women	Exploratory qualitative study	Incarcerated women over the age of 50 and/or with a chronic illness (n = 20) Mean age: 50 Males: 0% Females: 100%	- Semi-structured qualitative interviews - Grounded theory coding framework triangulated with fieldnotes from ethnographic observations for 18 months	Being locked in their cells for 23 h per day or more, often for days, weeks or even months, and the resulting isolation from loved ones, were detrimental to both their physical and mental health Access to mental healthcare for those in the general population was limited prior to the pandemic, and that COVID-19 risk mitigation strategies, including the cessation of group programs and shift to cell-front mental health services, created further barriers Without intervention, COVID-19 mitigation strategies may result in more incarcerated people developing anxiety, depression and other mental health conditions, or the exacerbation of these conditions among those with prior mental health diagnoses. There may be unique consequences for older populations and people of colour	9/10
Kothari et al. [42]	United Kingdom	To understand whether there had been changes in their mental health and what had contributed to this To understand the impact of the reduced regime and other changes To know whether those that required it were feeling supported by the prison minimal services of the mental health	Cross-sectional descriptive study	Prisoners of a male urban prison (n = 104) Mean age: 36 Males: 100% Females: 0%	- Mixed methods approach with a convergent parallel design - Semi-structured questionnaires	Prisoners and staff reported finding it hard to cope with changes and stressors associated with the pandemic Time spent locked in one’s cell with limited access to activities and support was associated with poor mental health outcomes, and salient themes emerged of feeling trapped, isolated and neglected The implementation of additional restrictions, within the already restricted prison environment, has had a significant negative impact on the mental health The effects of the pandemic appear to have heightened an already desperate need to consider the mental health and wellbeing of prisoners and prison staff which must be urgently addressed	6/8
Mendes et al. [43]	Portugal	To investigate the perceived fear of COVID-19 and the psychological impact of the pandemic in a sample of young adult male inmates	Quantitative cross-sectional study	Male inmates imprisoned for over 2 years (n = 60) Mean age: 21.9 Males: 100% Females: 0%	- Sociodemographic variables - DASS-21 - FCV-19 - BRCS.	Fear of COVID-19 was positively related to age, anxiety, and stress but negatively associated with the perception of mental health The final model was able to explain 49.7% of the total variance in fear of COVID-19, with mental health-related predictors explaining most of the variance High prevalence of stress among inmates, as well as moderate levels of anxiety and depression: 75%, 38.3%, and 36.7% of individuals, respectively, exhibited mild to extremely severe stress, anxiety, and depression symptoms	7/8
Montagnet et al. [46]	Northeastern United States	To explore the shifting conditions of confinement in the early months of the pandemic, as well as how incarcerated individuals experienced the pandemic in terms of their mental and physical health, safety, and trust in correctional institutions	Qualitative study	Individuals released from state prisons, county jails, and halfway houses (n = 53) Males: 90% Females: 10%	- Semi-structured interview	Lockdown within prisons led to limited contact with loved ones and anxiety about contracting the virus Three interrelated difficulties derived from lockdown measures: 1) Compromised contact with loved ones; combined with: 2) Anxiety about contracting COVID-19 (COVID-19 anxiety in prison) and: 3) Further eroded their trust in correctional institutions	7/10
Sorge et al. [47]	Italy	To explore the psychological effects of lockdown during the early stages of the COVID-19 pandemic on people living in an Italian prison	Qualitative study	Italian and foreign male prisoners detained in a Lombardy prison during the lockdown phase (n = 17) Mean age: 41.69 Males: 100% Females: 0%	- Qualitative content analysis of 27 posts, written by participants and published on the blog, concerning personal experiences on COVID-19	The analysis showed that the content of the publications was predominantly negative in emotional terms The most frequent negative emotional connotations coded were: loss, worry, psychological pain, and fear	7/10
Suhomlinova et al. [48]	United Kingdom	To explore how a socially excluded gender minority group —transgender and non-binary (TGNB) prisoners— experienced, and coped with, the pandemic stressors	Longitudinal qualitative study	13 transgender female and 2 non-binary participants, inmates in prisons of England and Wales (n = 15) Males: 14% (non-binary) Females: 86%	- Semi-structured questionnaire - Inductive and reflexive approach - Organic thematic analysis (Braun y Clarke, 2019)	TGNB prisoners experienced added stressors associated with their intersecting incarceration and gender minority positions, including prolonged solitary confinement and reduced access to gender-affirming healthcare Environmental resources for problem-focused, emotion-focused, socially supportive and disengagement coping were reduced by the prison pandemic regime, with adaptive coping through positive distraction and engagement with TGNB/LGBTQ community particularly affected	9/10
Wainwright et al. [49]	United Kingdom	How and in what ways prison healthcare changed in response to COVID-19 and how these changes were experienced	Inductive qualitative study	Prison leavers who had been in prison during the pandemic (12 men and 3 women) (n = 15) Males: 80% Females: 20%	In-depth interviews (over phone or video)	Reduced access and changes to how healthcare was delivered. This affected the health of prisoners by exacerbating existing conditions, new conditions being undiagnosed and mental health needs increasing Some participants described a sense of trauma, feelings of abandonment, frustration by the lack of face-to-face healthcare and the length of time spent behind their cell doors Solitary confinement can cause anger, depression, anxiety, paranoia, psychosis, and aggravate pre-existing mental illness	8/10
Zhang et al. [44]	China	To compare the anxiety levels in prisoners before and after the COVID-19 outbreak and analysed the causes of the changes in anxiety	Longitudinal study	Longitudinal cohort of 803 male inmates without serious mental disorder (480 without anxiety and 323 with anxiety) (n = 803) Mean age: 40.2 Males: 100% Females: 0%	- I survey X- 2019 and II in III- 2020 - GAD‐7 - PHQ ‐9 - Insomnia Severity Index scales	COVID-19 had a negative impact: it is a risk factor for anxiety among prisoners during the pandemic Prisoners who felt more anxious during the COVID-19 process were those without anxiety at the start of the study, but prisoners with previous anxiety felt less anxious Prisoners without anxiety, with fewer years of education, with COVID-19, and more severe depression and insomnia were associated with a new onset of anxiety COVID-19 was also associated with the onset and exacerbation of anxiety among prisoners, but the closed environment was familiar to them. Also, some changes in prison management were implemented after the COVID-19 outbreak, which may have reduced anxiety	6/8

AOR, adjusted odds ratio; BRCS, brief resilient coping scale; BSI 18, Brief Symptom Inventory 18; CI, confidence interval; COPE, coping orientation to problem experienced scale; DASS-21, Depression Anxiety and Stress Scale 21; FCV-19, Fear of COVID-19 Scale; GAD‐7, Generalised Anxiety Disorder ‐ no. of items; Oslo 3-item, Oslo 3-item questionnaire; PHQ 2-8-9, Patient Health Questionnaire–No. of items; SRH, Self-Rated Health; TGNB, Transgender And Non-Binary.

## Discussion

The aim of this study was to describe how COVID-19 affected the levels of different mental health variables in prison inmates. To this end, it attempted to provide a comprehensive response, not only based on key variables, but also by sequencing their structure and relationships in a factorial explanatory nexus with a global approach, within an aetiological framework, which also included differences in sex and gender.

In general, the mental health of prisoners has deteriorated over the pandemic [50]. As shown in the systematic review by Williams et al. [51], prisoners were generally found to have higher rates of SARS-CoV-2 infection, poorer clinical outcomes, and greater mental health deterioration during the pandemic than the general population.

On the one hand, based on the basic emotions found, such as fear of COVID-19, 49.7% of the variance was explained in one study [43], which can be justified as a natural response to a perceived threat. On the other hand, fear was also associated with complex emotions and cognitions that had been identified in studies, such as worry, psychological pain [47], abandonment, frustration [49], disturbing memories, and feeling isolated, left out, and insecure [42]. All of these factors can precipitate the response to fear, as they are perceived as threatening. Similarly, they may also be related to loneliness in women [41] due to the lack of attention received and a solitary confinement for the whole day, lasting for a long time [49]. In a complementary way, another study identified phenomena of alienation and worry, together with other emotions and problems when self-rating mood and emotions, indicating alexithymia [42]. These types of negative emotions have already been confirmed in other studies, namely, worry, psychological pain, fear [29], and fear of dying [49].

On the other hand, confinement may have worsened contact with relatives, levels of anxiety about contagion, and trust in professionals, both in incarcerated and recently released people, as is the case [46], as the convergence of these factors could involve cyclical relationships between psychological antecedents and consequences, given that most of the possible interactions took place with the professionals. In this sense, the inmates’ trust in the prison as an institution and in themselves in this respect was perceived as low [40]. Previous studies have already noted that low self-esteem, lack of confidence, uncertainty, a sense of survival, and hopelessness may precede the development of signs and symptoms of anxiety and depression. In addition, they may precede the cognitive triad that predicts possible depression, including negative thoughts about oneself, the world, and the future [52]. This would explain the results of the study, where worsening depression increased anxiety more than other related factors such as social support [39]. Affected self-esteem and confidence, linked to uncertainty and survival, may underlie anxiety and depression [52].

Depressive symptoms also increased during the pandemic [39, 40, 43], less likely in the absence of severe insomnia and in the presence of strong social support [37]. Also, substance use doubled the likelihood of developing depressive symptoms [37], given previous high dependence [42] perhaps exacerbated by difficulties in access, cancelled visits and releases, and fewer appointments for psychotropic medication, as reported in previous studies [53]. In other studies, inmates were found to have a mood that was categorised as average [38, 42], though rated as worse than in other groups. This may be a consequence of being immersed in uncontrollable events, generating a state of acquired helplessness. According to the Learned Helplessness theory, in the face of uncontrollable events, individuals reduce their responses because they expect them not to influence the consequences; they find it difficult to learn that the consequences depend on their responses, and with aversive consequences, behavioural and physiological disorders of anxiety and fear occur, followed by depression [54].

Regarding the anxiety found, it was often associated with distress, as observed in a study with women where 34.6% could be distressed due to the risk of contagion and to restrictions [41]. In men, they were moderately to severely distressed by fear of infection and, on the other hand, by uncertainty [43]. At a general level, distress was also detected along with anxiety, alienation, and pain, as it triggers negative emotions, anxiety, and depression [38], increases generalised anxiety [39] due to restrictions [41, 48], risk of infection [29, 38, 41, 43], as well as fear, uncertainty [43], and other negative emotions [38]. All of these factors may favour the persistence of the concern beyond the initial threat and may condition its severity, which could be related to the acquired helplessness mentioned above.

In women, and among common findings with men, it was found that the reduction in appointments made it more difficult to access mental healthcare, which was already limited [45], and anxiety was observed to increase when they were unable to have contact with deceased loved ones or infected family members and friends due to the suspension of visits [41]. Among transgender women and non-binary persons (TGNB), additional stressors such as solitary confinement for reasons of sex and gender and reduction of gender-affirming care, of social support from the TGNB community, and of specific mental healthcare were experienced [48].

Accordingly, in two studies, moderate anxiety was found in nearly the 40% of individuals [42, 43]. Generalised anxiety was identified at 51.7% in one study [40] and at 66.9% in another one, being more severe and persistent in case of disproportionate worry about infection and severe insomnia [37], all descriptive symptoms of generalised anxiety. Probably, the restrictive and overcrowded conditions in Ethiopia, where the study with the highest score was conducted, may explain these results compared to those found in Italy, also because of the different assessment tools used, GAD 2 and 7, respectively, the calculated size, and the characteristics of the sample. It is striking that in the study by Zhang et al. [44] in prisoners with a history of anxiety, this was reduced during the pandemic, and appeared in those with lower levels of education, with COVID-19, depression, or insomnia. This contrasts with the study in which symptoms were found to increase [39], and with another one where inmates noted that the decrease in routines affected their autonomous programming [42]. This could be improved, as recommended in previous studies, by facilitating prison phone calls [30, 31] and enabling appropriate distancing, clear communication, risk assessment, and mental healthcare [30].

Regarding suicide in the selected studies, and following the TGNB study by Suhomlinova et al. [48], restrictions led to higher suicidal and anxiety patterns, coinciding with results found in a study in men, which predicted insomnia, addictions, self-harm, and self-harm attempts [38]. However, in another study involving women [41], suicidal ideation was low, perhaps because the pandemic would have helped to value their life and their support role in the family. It is known that isolation can aggravate previous mental conditions [31] and increase self-harming and suicidal practices [9, 10], correlating with hopelessness [52], the main precipitant of suicide, and with the ultimate expression of acquired helplessness [55].

Among the strengths of this review are the wide coverage of types of studies, which allows for a broader view of the phenomenon under study, as well as the fact that it is a little-studied topic with a population group difficult to access. In addition, it should be added that a scientific methodology has been followed so that it can be replicated, and the quality of the studies finally selected has been assessed. Previous related reviews are based more on describing the restrictive conditions derived from the pandemic in prisons as predisposing and precipitating factors for the mental health of inmates. However, there are few studies that describe and explain such effects and relate them to those factors in a way that allows for prevention and promotion of people’s mental health in similar situations, especially among particularly vulnerable population groups.

At the study level, limitations exist due to the different methodologies used, which have not allowed a review with meta-analysis to be carried out.

### Limitations

The present study has a number of limitations. Firstly, despite the time that has elapsed since the start of the pandemic, few studies exist on how the mental health of people in prison has been affected.

On the other hand, a high heterogeneity of studies was found. This implied varying study phenomena, methodology, countries, types of centres, organisation of these centres, and protocols for COVID-19 intervention, which somewhat hindered comparison of these studies.

However, the studies were generally consistent in highlighting influencing variables such as increased access to spaces, social relations, support resources, and information on the pandemic. Thus, as the main strategy to reduce confounding factors, it was decided to include any type of primary study, whether quantitative, qualitative, or mixed, with proven quality.

### Conclusion

The mental health of prison inmates has been affected by the COVID-19 pandemic, with increased levels of stress, anxiety, fear, depression, and negative emotions and cognitions, all of which were related to the lack of availability of information and to the isolation that resulted in reduced social interactions, access to common spaces and support resources, especially mental health resources, fear of contracting the virus, and lack of trust in professionals and in themselves to be protected. Yet, protective strategies such as ensuring access to psychotropic medication and psychotherapeutic support are indeed proposed.

Further studies are needed to complement these findings and contribute to greater evaluation and intervention, especially among vulnerable populations, including the elderly, women, and transgender and non-binary individuals. In addition, there is a need to implement prevention and mental health promotion programmes for inmates, as generalisable practices both for restoring an adequate level of mental health among affected inmates and for better coping with other pandemics or similar crises.
