# Supplementary material for: Mental Health of Prison Inmates During the COVID-19 Pandemic: A Systematic Review
Source: Int J Public Health. 2024 Nov 21;69:1607166. doi: 10.3389/ijph.2024.1607166 (PMC11617144; doi:10.3389/ijph.2024.1607166)
Supplement: Supplementary file 1 [file Table1.DOCX]

Supplementary File 1. Terms used in the search (COVID-19 in Prisons, Spain, 2020-2024).

| **MeSH** | **Meaning** | **Terms** |
| --- | --- | --- |
| Prisons | Penal institutions, or places of long-term confinement for prisoners | Prison* OR Penitentiar* OR inmates or criminals or offenders or incarcerated people |
| Mental health | Emotional, psychological, and social well-being of an individual or group. | Mental health OR depression OR anxiety OR stress OR fear OR burnout |
| COVID-19 | A viral disorder generally characterised by high fever; cough; dyspnea; chills; persistent tremor; muscle pain; headache; sore throat; a new loss of taste and/or smell (see ageusia and anosmia) and other symptoms of a viral pneumonia. | COVID-19 |
